# Influencing Factors and Effects of Treatment on Quality of Life in Patients With Gastric Cancer—A Systematic Review

**DOI:** 10.3389/fpsyt.2021.656929

**Published:** 2021-07-01

**Authors:** Sophia Kristina Rupp, Andreas Stengel

**Affiliations:** ^1^Department of Psychosomatic Medicine and Psychotherapy, University Hospital Tübingen, Tübingen, Germany; ^2^Section Psychooncology, Comprehensive Cancer Center Tübingen Stuttgart, University Hospital Tübingen, Tübingen, Germany; ^3^Department for Psychosomatic Medicine, Charité Center for Internal Medicine and Dermatology, Charite-Universitätsmedizin Berlin, Corporate Member of Freie Universität Berlin, Humboldt-Universität zu Berlin and Berlin Institute of Health, Berlin, Germany

**Keywords:** anxiety, depression, gastric cancer, quality of life, psychiatric, psychooncology, psychosocial

## Abstract

**Background:** Gastric cancer (GC) is one of the leading causes of death worldwide. It is associated with several disease-related impairments contributing to the psycho-social burden of those patients, such as deterioration of well-being and overall quality of life (QOL). The aim of this study is to present the wide range of factors potentially impacting patients' overall well-being and possible preventive interventions.

**Methods:** This systematic review was conducted in October 2020 with a search in the PubMed, MedLine, PsycInfo, and Google Scholar databases. We used the keywords “gastric cancer,” “gastric neoplasm,” and each of them combined with “quality of life,” “depression,” and “anxiety” to identify all relevant articles reporting about potential impact factors influencing the overall well-being of patients suffering from gastric cancer.

**Results:** Finally, 125,490 articles were found, of which 125,431 were excluded in several steps of screening. Inclusion criteria were studies carried out on human ≥18 years of age, studies in English or German language, clinical trials, registry-based studies, cohort studies, population-based studies, and certain titles and abstracts. After screening for eligibility 35 potential factors influencing overall well-being in patients with GC were identified and classified into 9 important categories: genetic condition, treatment method, blood markers, nutritional status, daily living, state of health, mental state, supportive care, and alternative treatment.

**Conclusion:** Since various factors are involved in the development of patients' overall well-being, timely treatment of psycho-social impairments by physicians and psychologists is of enormous importance. Preventing psycho-social burden by improving patients' QOL should be of high importance in the treatment regimen of patients with GC.

## Introduction

GC is considered to be the worldwide most frequently seen type of cancer of the digestive system (1, 2). Moreover, it is one of the most prevalent form of cancers, and the third leading cause of cancer- related death (3, 4). In a previous epidemiological study, in 2018 more than 1,000,000 new cases and 783,000 deaths were reported globally (5, 6). Even though the numbers of reported new cases show a declining tendency, GC is still the second most common type of cancer after lung cancer (2).

At the early stage GC might present with characteristic symptoms including indigestion and stomach discomfort. Additionally, affected patients may struggle with bloated feeling after eating, mild nausea, loss of appetite, and heartburn (7). Patients in the advanced stage of GC often also suffer from blood in the stool, stomach pain, inexplicable weight loss, ascites, and vomiting (8). Besides physical symptoms, GC also leads to psychiatric symptoms/disorders (9). Here, anxiety and depression are reported as the most frequently encountered problems. Therefore, the mental health of affected patients should be regarded early on and during the whole course of treatment of GC (10). Additionally, there appears to be a correlation between cancer diagnosis and an increased risk for several mental disorders for patients with GC (11).

There are several treatments to cure, to prolong life and to relieve symptoms in patients with GC such as surgery, radiation, and chemotherapy. Considering the severity of these treatments and the mostly poor prognosis, patients with GC are of high risk to develop mental comorbidities (12). More precisely, these treatments have frequently shown short- and long-term effects resulting in a negative impact on health- related QOL. For instance, surgery as the standard curative treatment has an enormous effect on QOL and might result in distress (13). Patients that underwent gastrectomy are required to follow a specific restrictive diet. Loss of stomach storage capacity and pyloric sphincter functions, reflux, vitamin B12 deficiency, and weight loss are commonly reported even after successful treatment of GC (14). Furthermore, patients with GC may suffer from anxiety considering possible recurrence and a lack of capability to fulfill their usual tasks in household, workplace, and social life after treatment. Moreover, they may also experience the burden of the stigma by their family, friends, or colleagues possibly perceiving them to be weak and less resilient (15). Therefore, besides treatment and cure, the state of patients' QOL has received increasing attention. This is also the case with regard to clinical decisions. For instance, several QOL variables are an important prognostic factor for survival in stomach cancer. To provide this information to physicians, the impact of the disease on physical, social, and emotional health needs to be evaluated. Clinicians should keep in mind that timely treatment of psycho-social impairments by physicians and psychologists is of enormous importance. Subsequently, preventing psycho-social burden by improving patients' QOL should be of high importance in the treatment regimen of patients with GC. Therefore, health- related QOL of patients with GC should be assessed using validated questionnaires (16).

Since there is no universal definition of QOL, more precisely health- related QOL, questionnaires focus on the evaluation of positive, and negative experiences made by patients with GC. This assessment is mostly performed in considering at least 4 dimensions such as physical, emotional, and social functions, as well as the symptoms that are related to the disease and its treatment. Additionally, other dimensions such as spiritual well-being or sexual function recently gained more attention. Referring to the fact that there is evidence that one's QOL plays an important role as a prognostic factor, especially in metastatic disease, QOL should always be taken into account (13).

In accordance with the PICOS scheme, we focused on human studies of any type (study design) investigating patients with GC (population) with symptoms of psychosocial burden or decreased overall well- being (intervention), where applicable in comparison to patients with other interventions, cancer types, or healthy controls (comparison) regarding prevalence, magnitude, and associations of psychosocial burden and overall well-being (outcome).

Taken together, in this review we will focus on the various factors that may have an impact on the QOL of patients with GC to raise awareness for this important topic and therefore, help clinicians to potentially counteract the deterioration in the overall well-being of patients with GC.

## Search Methods

### Search Strategy

We followed the preferred reporting items for systematic reviews and meta-analyses (PRISMA) statement to report the results of this review. This article is a systematic review; articles related to the topic, were searched, collected, and screened. Articles released from the earliest day of publication to the day the search was performed were included. The last search was conducted on 16th October in 2020. We searched Pubmed, MedLine, PsycInfo, and Google Scholar using the following keywords: “gastric cancer,” “gastric neoplasm,” and each of them combined with “depression,” “quality of life,” and “anxiety” (Table 1). All studies that contained material applicable to the topic were considered. Retrieved manuscripts were reviewed by the author and were extracted using a standardized collection tool.

**Table 1:** The search strategy of the research.

**Search engines and databases**	**•PubMed •MedLine •PsycInfo •Google Scholar**
**Search date**	**Up to 2020, October, 16**
**Search terms**	**Strategy: #1 AND #3, #1 AND #4, #1 AND #5, #2 AND #3, #2 AND #4, #2 AND #5 •#1 „Gastric cancer” •#2 „Gastric neoplasm” •#3 „Quality of life” •#4 „Depression” •#5 „Anxiety”**

### Inclusion and Exclusion Criteria

The inclusion criteria were as follows: (1) Clinical trials, registry-based studies, cohort studies, population-based studies; (2) Studies that included adults ≥18 years of age; (3) Studies that included patients with GC; and (4) Studies that were written in English or German.

The exclusion criteria were as follows: (1) Review articles, meta- analyses, surveys, case reports, comments, letters, conference abstracts or posters, or economic evaluation; (2) Studies including children <18 years of age; (3) Studies in which abstracts, or full-text articles were not available; (4) Studies that were not available in English or German; (5) Studies that included patients with additional other cancer types (e.g., esophageal cancer) and; (6) Studies reporting about extremely specific drug treatment variations or were Phase I or Phase II drug trials.

### Study Selection and Data Extraction

One reviewer screened all titles and abstracts retrieved from the electronic searches to identify potentially eligible articles. Full texts of the potentially eligible articles were retrieved. The same reviewer screened all full-text articles and screened potentially eligible or unclear full-text articles, determined whether they were eligible or not eligible and recorded the reason for exclusion.

The quality of the studies included in this review was assessed with respect to risk of bias within individual and across studies by thoroughly evaluating the study designs, selection of participants, methodological procedures applied as well as presentation of the results.

Following a full-text review of the eligible studies, one reviewer extracted the relevant data. From each included study the following information was extracted: first author of the publication, year of publication, country, number of reported cases, research question/purpose, the method used, and key findings.

## Results

**Figure 1:**
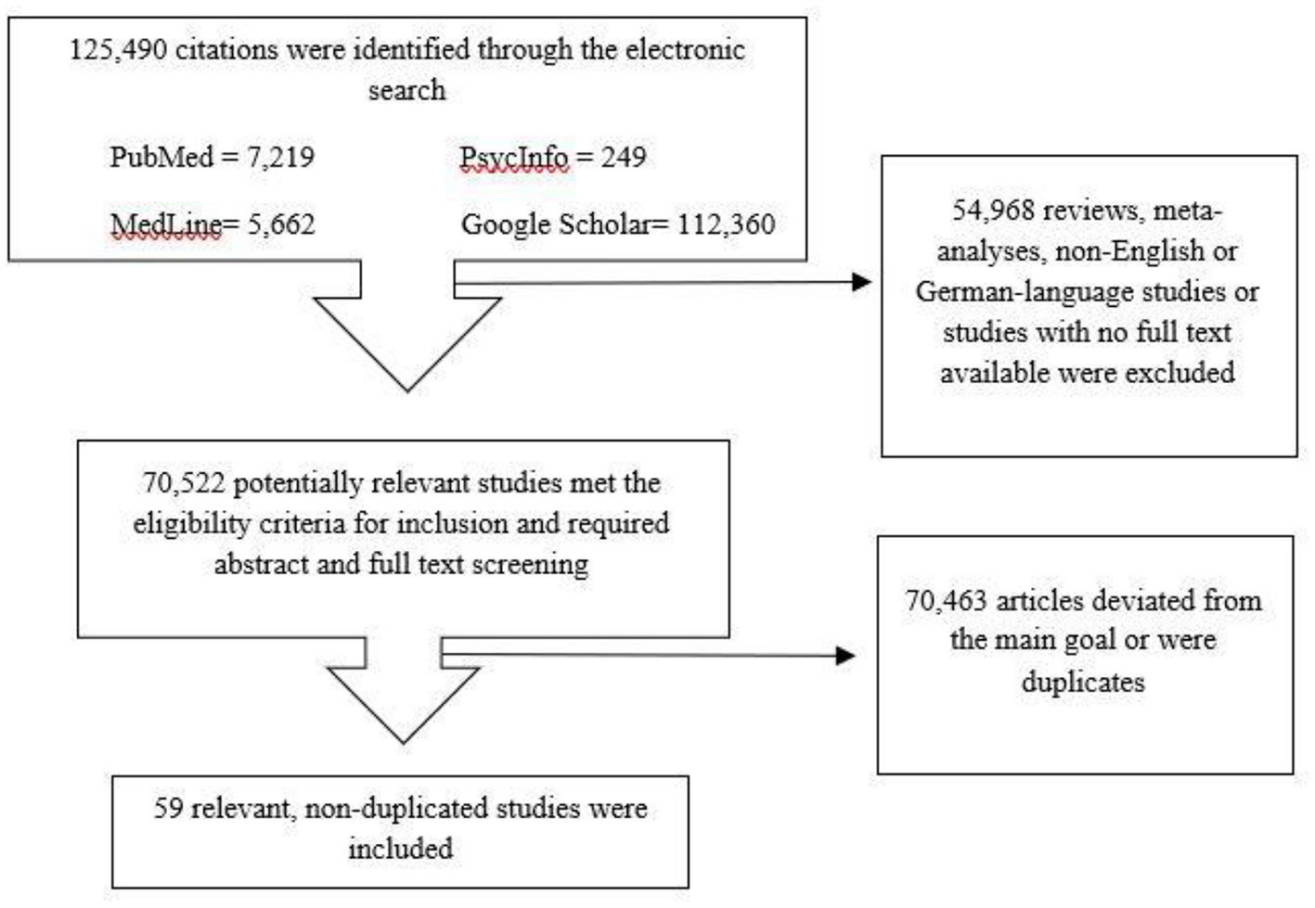
Flowchart for article screening and selection.

One hundred twenty-five thousand four hundred ninety articles were identified after searching the databases using the keywords mentioned above. Reviews, meta- analyses, surveys, case reports, comments, letters, conference abstracts or posters, or economic evaluation and non-English- or German- language studies or studies with no full text available, were excluded and the number decreased to 70,522 articles. Next, studies not related to the review topic (e.g., studies with patients suffering from esophageal carcinoma), or studies reporting about extremely specific drug treatment variations or were Phase I or Phase II trials and duplicates were excluded. Ultimately, 59 articles were selected for this systematic review. The PRISMA flow diagram schematically depicts the article selection process (Figure 1). Table 2 shows the main results of these articles. The description of the study populations is provided in Table 3.

**Table 2:** Main results of articles discussed in this systematic review (in order of discussion).

**First author**	**Title**	**Year**	**Country**	**Main results**
Koh et al. (17)	Influence of the BDNF Val66Met polymorphism on coping response to stress in patients with advanced gastric cancer	2014	Korea	Met carriers of BDNF Val66Met polymorphism may be a predictive factor for an anxious coping response in patients with GC.
Kang et al. (18)	FKBP5 polymorphisms as vulnerability to anxiety and depression in patients with advanced gastric cancer	2012	Korea	There are special polymorphisms of FK506-binding protein (FKBP5) that are potent predictive factors for anxiety and depression after prolonged stress exposure due to treatment of GC.
Heydarnejad et al. (19)	Factors affecting quality of life in cancer patients undergoing chemotherapy	2011	Iran	Cancer type, pain intensity and fatigue are linked to each other. Patients who finished 3–5 chemotherapy cycles (CT) show higher QoL than patients with <2 CT cycles.
Park et al. (20)	Quality of life in patients with advanced gastric cancer treated with second-line chemotherapy	2006	Korea	Patients treated with second-line chemotherapy show an improvement in terms of global health/QoL, emotional function, cognitive function, and symptoms.
Kim et al. (4)	Treatment patterns and changes in quality of life during first-line palliative chemotherapy in Korean patients with advanced gastric cancer	2019	Korea	Patients' QoL is nearly maintained, regardless of their actual response to chemotherapy.
Cui et al. (21)	Combined cellular immunotherapy and chemotherapy improves clinical outcome in patients with gastric carcinoma	2015	China	Chemotherapy combined with cellular immunotherapy improves patients' QoL and leads to a longer progression-free survival period.
Kassam et al. (22)	Evaluating the impact on quality of life of chemoradiation in gastric cancer	2010	Canada	Global QoL is worsening during chemoradiation and is also influenced by the chemotherapy dose.
Kim et al. (23)	Safety and efficacy of fast-track surgery in laparoscopic distal gastrectomy for gastric cancer	2012	Korea	Fast-track surgery in laparoscopic distal gastrectomy in patients with GC improves immediate postoperative QoL and accelerates postoperative recovery.
Lee et al. (24)	Quality of life beyond the early postoperative period after laparoscopy-assisted distal gastrectomy: the level of patient expectation as the essence of quality of life	2012	Korea	Patients who underwent laparoscopy-assisted distal gastrectomy experience lower QoL compared to patients who underwent open distal gastrectomy after the early postoperative period and before achieving long-term survival. These results may be associated with the patients' expectation.
Kim et al. (25) Misawa et al. (26) Lee et al. (27)	Improved quality of life Outcomes after laparoscopy-assisted distal gastrectomy for early gastric cancer Long-term quality of life after laparoscopic distal gastrectomy for early gastric cancer What is the best reconstruction method after distal gastrectomy for gastric cancer?	2008 2015 2012	Korea Japan Korea	Laparascopy-assisted distal gastrectomy does not only lead to less pain, recovery etc. but also improves patients' QoL in terms of global health and patient functioning compared to open operation.
Park et al. (28)	Comparison of laparoscopic proximal gastrectomy with double-tract reconstruction and laparoscopic total gastrectomy in terms of nutritional status or quality of life in early gastric cancer patients	2018	Korea	Regarding patients' QoL, there is no significant difference between patients who have undergone laparoscopic proximal gastrectomy (LPG) and those who have undergone laparoscopic total gastrectomy (LTG) during the follow-up period of 2 years, except for physical function.
Fujita et al. (29) Kim et al. (30)	Assessment of postoperative quality of life following pyloruspreserving gastrectomy and Billroth-I distal gastrectomy in gastric cancer patients: results of the nationwide postgastrectomy syndrome assessment study A randomized controlled trial of vagus nerve-preserving distal gastrectomy vs. conventional distal gastrectomy	2016 2016	Japan Korea	Preservation of the celiac branch of the vagus nerve leads to a higher QoL compared with a conventional distal gastrectomy.
Ikeguchi et al. (31)	A new pouch reconstruction method after total gastrectomy (pouch-double tract method) improved the postoperative quality of life of patients with gastric cancer	2011	Japan	Pouch-double tract method (PDT) after total gastrectomy (TG) improves patients' nutritional conditions more than reconstruction with traditional Roux-en-Y reconstruction (RY). Thus, PDT might improve QoL of patients who underwent TG.
Rutegard et al. (32)	Determinants of global quality of life before and after major cancer surgery	2009	United Kingdom	The main predictors of global QoL in patients with GC differ before and after major cancer surgery. Before surgery the main predictors are physical and emotional function while after surgery fatigue and dyspnea are the strongest predictors of global QoL.
Davies et al. (33) Gockel et al. (34)	Total or subtotal gastrectomy for gastric carcinoma? A study of quality of life Quality of life after subtotal resection and gastrectomy for gastric cancer	1998 2004	United Kingdom Germany	Patients who had undergone subtotal gastrectomy (SG) show a higher QoL after operation than before surgery and 1 year postoperatively compared to patients who underwent TG.
Goh et al. (35)	Quality of life after total and subtotal gastrectomy for gastric carcinoma	2014	United Kingdom	There are no significant differences in overall QoL in patients after SG and TG. The parameters “dysphagia” and “eating restrictions” were higher in patients with TG.
Yu et al. (36) Avery et al. (37)	Chronological changes of quality of life in long-term survivors after gastrectomy for gastric cancer Health-related quality of life and survival in the 2 years after surgery for gastric cancer	2016 2010	Korea United Kingdom	Gastrectomy for cancer has an enormous decreasing effect on QoL.
Zieren et al. (38)	Quality of life after surgical treatment of gastric carcinoma	1998	Germany	Postoperative QoL is affected mainly by somatic complaints and physical limitations. Cancer recurrence is the decisive factor in deciding patients' QoL.
Arner et al. (39)	Circulating carnosine dipeptidase 1 associates with weight loss and poor prognosis in gastrointestinal cancer	2015	Sweden	Patients with GC and a lower serum level of circulating carnosine dipeptidase 1 tend to have a higher weight loss, malnutrition and poor QoL.
Fujita et al. (40)	Circulating alpha-2-macroglobulin levels and depression scores in patients who underwent abdominal cancer surgery	2003	Japan	Circulating A2M elevation plays a role in the development of postoperative depression in patients with. Especially patients who underwent total gastrectomy are predisposed to depression.
Pan et al. (41)	Leptin-LepRb expressed in gastric cancer patients and related to cancer-related depression	2017	China	Both serum and tissue Leptin-LepRb are higher in depressive patients with GC than in non-depressive patients with GC.
Xu et al. (10)	Prevalence rate and influencing factors of preoperative anxiety and depression in gastric cancer patients in china	2016	China	The neutrophil-to-lymphocyte ratio (NLR) is a factor influencing the prevalence of preoperative anxiety and depression of patients with GC.
Correia et al. (42)	Serum concentrations of TNF-alpha as a surrogate marker for malnutrition and worse quality of life in patients with gastric cancer	2007	Portugal	Cytokines, more precisely TNF-alpha, can be specifically associated to malnutrition along with worse QoL in patients with GC.
Wei et al. (43) Huang et al. (44)	Oxidative stress in depressive patients with gastric adenocarcinoma ROS are involved in the development of gastric cancer and gastric cancer-related depression through ABL1-mediated inflammation signaling pathway	2009 2019	China China	Oxidative imbalance and oxidative stress in depressive patients with GC influence the onset and exacerbation of depression.
Tian and Chen (45) Park et al. (46) Climent et al. (47)	Nutritional status and quality of life of the gastric cancer patients in Changle County of China Impact of body mass index on the quality of life after total gastrectomy for gastric cancer	2005 2018 2017	China Korea Spain	Daily nutrition intake is positively correlated with QoL in patients with GC.
	Weight loss and quality of life in patients surviving 2 years after gastric cancer resection			
Hur et al. (48)	Effect of early oral feeding after gastric cancer surgery	2011	Korea	Early oral feeding after gastric cancer surgery results in shorter hospitalization and improves QoL in terms of less fatigue and less nausea in the early postoperative period.
Jin et al. (49)	Effects of post-surgical parenteral nutrition on patients with gastric cancer	2018	China	Post-surgical parenteral nutrition improves the nutritional and psychological status, QoL and immune function of patients with GC after surgery.
Tian et al. (50)	Comparison of quality of life between urban and rural gastric cancer patients and analysis of influencing factors	2003	China	QoL of patients living in rural areas are worse compared to the QoL of patients living in urban areas. Patients having higher family income score higher in QoL. Enhanced nutrition and rehabilitating exercise positively influence the QoL of patients with GC.
Wang et al. (51) Kim et al. (52)	The quality of life of Chinese middle-aged male patients with gastric carcinoma after total gastrectomy and nursing intervention Prevalence and prognostic implications of psychological distress in patients with gastric cancer	2010 2017	China Korea	There is a negative association between patients' QoL and a higher education level and economic condition.
Faller et al. (53)	Effectiveness of education for gastric cancer patients	2009	Germany	Patients attending an interactive education program regarding information about their disease had more knowledge, showed more active coping and reported a better QoL.
Dang et al. (54)	Quality of life in Vietnamese gastric cancer patients	2019	Vietnam	Disruption of sexual activity and difficulty in maintaining daily activities are two frequent and serious adverse conditions of GC and its treatment. Educational status, age, occupation, disease stage, treatment method, and time from diagnosis are additional factors that affect health-related QoL of patients with GC.
Hofheinz et al. (55)	Patient preferences for palliative treatment of locally advanced or metastatic gastric cancer and adenocarcinoma of the gastroesophageal junction	2016	Germany	Patients with GC consider a survival benefit accompanied by high QoL in terms of being able to self-care and receiving a treatment with good tolerability as more important than only an additional survival benefit.
Bilgin and Gozum (2)	Effect of nursing care given at home on the quality of life of patients with stomach cancer and their family caregivers' nursing care	2018	Turkey	Nursing care provided during home visits to the patients in the experimental group improves patients' overall QoL, global health conditions, emotional functions and symptoms related to the disease.
Jeong and An (56)	The moderating role of social support on depression and anxiety for gastric cancer patients and their family caregivers	2017	Korea	Social support and patients' income have an influence on their anxiety.
Hu et al. (57)	Depressive disorders among patients with gastric cancer in Taiwan	2018	Taiwan	Female sex and hypertension are predictive variables for the development of a depression in patients with GC.
Huang et al. (58)	Effect of surgery-induced acute muscle wasting on postoperative outcomes and quality of life	2017	China	Acute muscle wasting negatively affects QoL of patients with GC.
Lee and Lim (15)	Mediation effect of adaption on the quality of life in patients with gastric cancer undergoing gastrectomy	2019	Korea	Adaptation to the diagnosis, followed by anxiety, perceived gastrointestinal symptoms, social support, spiritual well-being and self-efficacy are important factors that influence QoL of patients with GC.
Baudry et al. (12)	The role of trait emotional intelligence in quality of life, anxiety and depression symptoms after surgery for esophageal or gastric cancer: A French national database FREGAT	2019	France	Intrapersonal and interpersonal emotional competence (EC) after diagnosis and surgery predict fewer anxiety and depression symptoms of patients and better health-related QoL.
Yamaoka et al. (59) Zhang et al. (60)	Health-related quality of life varies with personality types Type D personality in gastric cancer survivors: association with poor quality of life, overall survival, and mental health	1998 2016	Japan China	The QoL level depends on the patients' personality type.
Nordin et al. (61)	Predicting anxiety and depression among cancer patients	2001	Sweden	Patients' level of depression and anxiety at diagnosis is a predictor of depression and anxiety 6 months later. Other risk factors at diagnosis are advanced disease and a lack of somebody besides the family to rely on in difficult situations regarding the disease.
Ben-Ezra et al. (62)	The association between previous psychological trauma and mental health among gastric cancer patients	2011	Israel	Patients with GC who experienced previous trauma show higher depressive symptoms, lower perceived social support and lower future life satisfaction. Gender and marital status are predictors of psychiatric symptoms, while age, marital status and past life satisfaction are predictors of well-being.
Song et al. (63)	Waiting time for cancer treatment and mental health among patients with newly diagnosed esophageal or gastric cancer: a nationwide cohort study	2017	Sweden	Patients without any previous mental disorder benefit from longer waiting times for cancer treatment regarding their future mental health while patients with previous mental disorders profit from a quicker treatment decision.
Fukui et al. (64) Rha et al. (65) Fukui et al. (66)	Effectiveness of communication skills training of nurses on the quality of life and satisfaction with healthcare professionals among newly diagnosed cancer patients Unmet needs in the physical and daily living domain mediates the influence of symptom experience on the quality of life of gastric cancer patients A randomized study assessing the efficacy of communication skill training on patients' psychologic distress and coping	2008 2020 2011	Japan Korea Japan	Improved communication skills by nurses lower psychologic stress among patients after being informed of a cancer diagnosis.
Hafizi et al. (67)	A randomized, double-blind, placebo-controlled investigation of BCc1 nanomedicine effect on survival and quality of life in metastatic and non-metastatic gastric cancer patients	2019	Iran	BCc1 nanomedicine improves patients' QoL and leads to higher overall survival.
Zhan et al. (68)	Clinical study on safety and efficacy of Qinin® (Cantharidin Sodium) injection combined with chemotherapy in treating patients with gastric cancer	2012	China	Cantharidin sodium injection combined with chemotherapy shows a positive impact on clinical benefit response, QoL, and reduces side effects of chemotherapy.
Sun et al. (69)	Therapeutic effect of Jinlongshe Granule (金龙蛇颗粒) on quality of life of stage IV gastric cancer patients using EORTC QLQ-C30	2015	China	Additional use of Jinlongshe Granule has a positive impact on the somatic function, role function, emotional function, social function, cognitive function, and general QoL of patients with advanced GC, and lowers the symptoms of fatigue, nausea and vomiting, pain, loss of appetite, and constipation
Kim et al. (70)	Quality of life, immunomodulation and safety of adjuvant mistletoe treatment in patients with gastric carcinoma—a randomized, controlled pilot study	2012	Korea	Adjuvant mistletoe treatment in patients with GC leads to less frequently diarrhea and is associated with improved QoL.

*GC, gastric cancer; QoL, quality of life*.

**Table 3:** Description of study populations discussed in this systematic review (in order of discussion).

**First author**	**Sample size**	**Instruments**	**Mean age ± SD (if indicated)**	**Population**
Koh et al. (17)	91	ECOG HAD MAC	57.7 ± 11.4	67.7% male, 32.3% female
Kang et al. (18)	93	ECOG HAD MAC	58.1 ± 11.6	67.7% male, 32.3% female
Heydarnejad et al. (19)	200	EORTC QLQ-C30	46.2	54.5% male, 45.5% female
Park et al. (20)	43	EORTC QLQ-C30 HAD	59	70% male, 30% female
Kim et al. (4)	527	EORTC QLQ-C30 QLQ-STO22	60	72.1% male, 27.9% female
Cui et al. (21)	58	EORTC QLQ-C30 QLQ-STO22	58.5	70.6% male, 29.4% female
Kassam et al. (22)	33	EORTC QLQ-C30	56	55% male, 45% female
Kim et al. (23)	44	EORTC QLQ-C30 QLQ-STO22	Intervention group: 52.64 ± 11.57 Control group: 57.45 ± 14.54	63.6% male, 36.4% female
Lee et al. (24)	80	EORTC QLQ-C30 QLQ-STO22	55.8 ± 10.4	53.7% male, 46.3% female
Kim et al. (25)	164	EORTC QLQ-C30 QLQ-STO22	55.6	60.3% male, 39.7% female
Misawa et al. (26)	145	EORTC QLQ-C30 QLQ-STO22	62.5	67.5% male, 32.5% female
Lee et al. (27)	159	GQLI	Open: 58.6 ± 10.6 Laparoscopy: 60.1 ± 11.4	Not indicated
Park et al. (28)	80	EORTC QLQ-C30 QLQ-STO22	64.2	68.6% male, 31.4% female
Fujita et al. (29)	2,368	PGSAS-45	61.6 ± 9.1	6.,5% male, 36.5% female
Kim et al. (30)	163	EORTC QLQ-C30 QLQ-STO22	55.2	77.2% male, 22.8% female
Ikeguchi et al. (31)	29	None	RY reconstruction: 69.5 ± 7.9 PDT reconstruction: 69.0 ± 13.7	68.9% male, 31.1% female
Rutegard et al. (32)	145	EORTC QLQ-C30	65.1	71.3% male, 28.7% female
Davies et al. (33)	47	HAD Troidl QOL	69	72.9% male, 27.1% female
Gockel et al. (34)	73	GQLI	71.9	62.7% male, 37.3% female
Goh et al. (35)	53	EORTC QLQ-C30 QLQ-STO22	73	56.6% male, 43.4% female
Yu et al. (36)	254	EORTC QLQ-C30 QLQ-STO22	54.9 ± 10.7	62.6% male, 37.4% female
Avery et al. (37)	58	QLQ-STO22	71	72.4% male, 27.6% female
Zieren et al. (38)	71	EORTC-QLQ-C36	59	66.1% male, 33.9% female
Arner et al. (39)	59	SF-36	64.2 ± 8.8	72.8% male, 27.2% female
Fujita et al. (40)	50	HAD	61	61.1% male, 38.9% female
Pan et al. (41)	105	DSM-4	Not indicated	66.6% male, 33.4% female
Xu et al. (10)	53	DS-14 HAD SSRS	59.0 ± 10.4	66% male, 34% female
Correia et al. (42)	48	EORTC QLQ-C30	69 ± 12	75.5% male, 24.5% female
Wei et al. (43)	106	HAMD SCL-90 SDS	52.5 ± 13.8	55.6% male, 44.4% female
Huang et al. (44)	110	DSM-5	Not indicated	69% male, 31% female
Tian et al. (45)	285	Self-generated questionnaire	Not indicated	Not indicated
Park et al. (46)	276	EORTC QLQ-C30 QLQ-STO22	59.2 ± 11.1	70.7% male, 29.3% female
Climent et al. (47)	76	EORTC QLQ-C30 QLQ-STO22	Not indicated	58.4% male, 41.6% female
Hur et al. (48)	58	EORTC QLQ-C30 QLQ-STO22	Not indicated	61.1% male, 38.9% female
Jin et al. (49)	108	EORTC QLQ-C30 HAD PHQ-9 SSLQ	Intervention group: 63.6 ± 5.2 Control group: 62.4 ± 5.3	73.7% male, 26.3% female
Tian et al. (50)	362	Self-generated questionnaire	Not indicated	85.3% male, 14.7% female
Wang et al. (51)	162	EORTC QLQ-C30	Not indicated	Not indicated
Kim et al. (52)	229	CES-D HAD MDT	56	72.9% male, 27.1% female
Faller et al. (53)	121	GQLI	61.4 ± 11.2	63.6% male, 36.4% female
Dang et al. (54)	182	15D	60.8 ± 11.6	Not indicated
Hofheinz et al. (55)	55	Self- generated questionnaire	63	78.2% male, 21.8% female
Bilgin and Gozum (2)	72	CQOLC EORTC QLQ-C30	Intervention group: 58.73 ± 10.86 Control group: 60.26 ± 12.05	48.6% male, 51.4% female
Jeong and An (56)	52	HAD FSSQ s	54.3 ± 12.1	57.7% male, 42.3% female
Hu et al. (57)	57,506	None	69	67.6% male, 32.3% female
Huang et al. (58)	110	EORTC QLQ-C30 QLQ-STO22	63.2 ± 10.4	73.6% male, 26.4% female
Lee and Lim (15)	297	EORTC QLQ-C30 HAD	63.1	64.3% male, 35.7% female
Baudry et al. (12)	228	EORTC QLQ-C30 HAD	63.5 ± 10.4	75% male, 25% female
Yamaoka et al. (59)	828	HRQOL-20	58.9 ± 11.8	48.1% male, 51.9% female
Zhang et al. (60)	830	EORTC QLQ-C30 HRQOL-20	Not indicated	53.4% male, 46.6% female
Nordin et al. (61)	522	HAD IES	64	39% male, 61% female
Ben- Ezra et al. (62)	123	GHQ PCLC SAS	57.3 ± 12.7	56.9% male, 43.1% female
Song et al. (63)	7,080	None	71.4 ± 11.9	65.7% male, 34.3% female
Fukui S (64)	86	SF-8	Intervention group: 61.4 ± 10.8 Control group: 60.9 ± 14.3	40% male, 60% female
Rha et al. (65)	223	EORTC QLQ-C30 HAD SCNS-SF 34	55.9 ± 11.7	59.2% male, 40.8% female
Fukui et al. (66)	89	HAD MAC	Intervention group: 61.4 ± 10.8 Control group: 60.9 ± 14.3	39% male, 61% female
Hafizi et al. (67)	123	QLQ-STO22	Intervention group: 59.8 ± 13.0 Control group: 61.2 ± 12.93	68.2% male, 31.8% female
Zhan et al. (68)	70	KPS score	Not indicated	Not indicated
Sun et al. (69)	39	EORTC QLQ-C30 KPS score	Not indicated	66.6% male, 33.4% female
Kim et al. (70)	32	EORTC QLQ-C30 QLQ-STO22	Intervention group: 53.7 ± 10.2 Control group: 54.8 ± 11.5	Not indicated

*CES-D, Center for epidemiologic studies depression scale; CQOLC, Caregiver quality of life index cancer scale; DS-14, the type D scale-14; DSM-4, Diagnostic and statistical manual of mental disorders- fourth edition; ECOG, Eastern cooperative oncology group score; EORTC-QLQ-C30, European organization for research and treatment of cancer quality of life core questionnaire; EORTC-QLQ-C36, European organization for research and treatment of cancer quality of life core questionnaire 36 items; EORTC- QLQ-STO22, European organization for research and treatment of cancer quality of life questionnaire-stomach; FSSQ, functional social support questionnaire; GHQ, general health questionnaire; GQLI, Gastric quality of life index; HAD, Hospital anxiety and depression scale; HAMD, Hamilton rating scale for depression; HRQOL-20, health- related quality of life questionnaire; IES, impact of events scale; KPS, score- Karnofsky performance status scale; MAC, Mental adjustment to cancer scale; MDT, modified distress thermometer; PCLC, posttraumatic stress disorder and depression checklist—Civilian Version; PGSAS-45, Postgastrectomy syndrome assessment scale; PHQ-9, Patient health questionnaire; SAS, Cantril's self-anchoring scale; SCL-90, symptom checklist-90; SCNS-SF 34, Supportive care needs survey; SDS, Sheehan disability scale; SF-8, Short-form health survey; SF-36, 36-item short form health survey; SSLQ, self- rating scale of life quality; SSRS, Suicide severity rating scale; Troidl QOL, Troidl quality of life index; 15D, quality of life questionnaire- 15 dimensional*.

### Quality Assessment

The current review consists of randomized studies or studies with a prospective or retrospective study design, assuming a risk for bias. Due to the fact that almost all studies used self-reporting questionnaires to assess symptoms, the risk of response bias was the most frequent type of bias. Furthermore, recall bias might have occurred as patients suffering from psychosocial burden might be more aware of it. Selection bias, which was also common, especially resulted from self-selection, as individuals suffering from specific symptoms might be more likely to return a questionnaire. Performance bias can be assumed in studies applying instrument-based techniques by a not blinded investigator.

**Figure 2:**
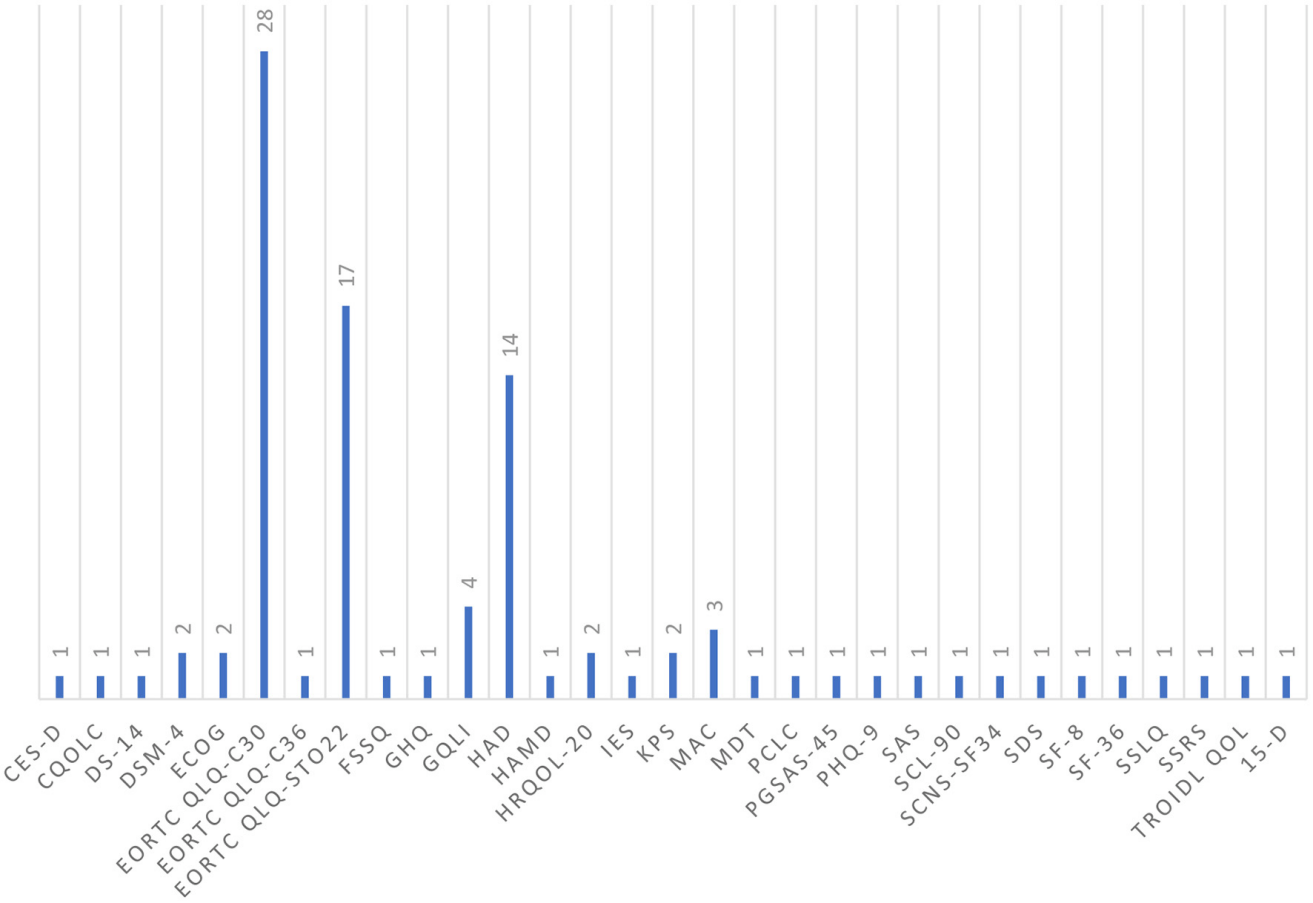
Overview of different psychometric instruments used in the studies discussed (in alphabetical order). CES-D, Center for epidemiologic studies depression scale; CQOLC- Caregiver quality of life index cancer scale; DS-14, the type D scale-14; DSM-4, Diagnostic and statistical manual of mental disorders- fourth edition; ECOG, Eastern cooperative oncology group score; EORTC-QLQ-C30, European organization for research and treatment of cancer quality of life core questionnaire; EORTC-QLQ-C36, European organization for research and treatment of cancer quality of life core questionnaire 36 items; EORTC- QLQ-STO22, European organization for research and treatment of cancer quality of life questionnaire-stomach; FSSQ, functional social support questionnaire; GHQ, general health questionnaire; GQLI, Gastric quality of life index; HAD, Hospital anxiety and depression scale; HAMD, Hamilton rating scale for depression; HRQOL-20, health- related quality of life questionnaire; IES, impact of events scale; KPS, score- Karnofsky performance status scale; MAC- Mental adjustment to cancer scale; MDT, modified distress thermometer; PCLC, posttraumatic stress disorder and depression checklist, Civilian Version; PGSAS-45-Postgastrectomy syndrome assessment scale; PHQ-9, Patient health questionnaire; SAS, Cantril's self-anchoring scale; SCL-90, symptom checklist-90; SCNS-SF 34, Supportive care needs survey; SDS, Sheehan disability scale; SF-8, Short-form health survey; SF-36- 36-item short form health survey; SSLQ, self- rating scale of life quality; SSRS, Suicide severity rating scale; Troidl QOL, Troidl quality of life index; 15D- quality of life questionnaire- 15 dimensional.

There are several issues that may contribute to limited comparability between studies and thus limited transferability of study results to the general population. First, the studies different instruments to assess quality of life, mental status and psychosocial burden or general overall well-being, either in the form of clinical interviews and/or based on questionnaires which may be a reason for the observed different prevalence rates. The wide range of questionnaires used to assess patients' QOL leads to hampered comparability of the studies. Even though, more compact questionnaires might save time and effort for both researchers and patients by assessing only about specific areas, they may yield to inaccurate results. Therefore, it shows the need for standardized questionnaires that are more comprehensive by shedding light on the variety of aspects that might influence patients' overall well- being. Figure 2 illustrates the wide range of instruments used in the studies discussed in this review. Second, the cancer stages, inclusion and exclusion criteria applied, and study populations used were very heterogeneous. In addition, clinical and therapeutic management and diagnostic investigations varied. Besides, some studies did not include a control group. Taken together, bias cannot be ruled out for the studies included in this systematic review; thus, these limitations should be kept in mind when interpreting the results discussed here.

According to the objectives of the present review and the examined studies, 35 influencing factors for psycho-social burden along with possible preventive interventions in patients with GC were identified and classified into nine main sections including genetic condition (2 factors), treatment method (7 factors), blood markers (6 factors), nutritional status (2 factors), daily living (5 factors), state of health (4 factors), mental state (4 factors), supportive care (1 factor), and alternative treatment (4 factors) (Table 4).

**Table 4:** Factors potentially influencing psycho-social burden (in order of discussion).

**Category**	**Factor**
Genetic condition	•BDNF Val66Met polymorphism •FKBP5 polymorphism
Treatment method	•Chemotherapy cycles and dose •Chemotherapy combined with radiotherapy, immunotherapy •Fast- track surgery •Laparoscopic- assisted, open gastrectomy •Preservation of the vagus nerve •Reconstruction method •Subtotal, total gastrectomy
Blood markers	•Carnosine dipeptidase 1 serum level •Circulating A2M serum level •Leptin-LepRb serum level •Neutrophil-to-lymphocyte ratio •TNF-alpha serum level •Oxidative stress balance
Nutritional status	•Daily intake •Post- surgical nutrition
Daily living	•Area of residence •Education status •Level of income •Nursing care at home •Social support
State of health	•Blood pressure •Muscle state •Severity of symptoms •Sex
Mental state	•Adaption, coping strategies •Emotional competence •Personality type •Previous mental state
Supportive care	•Communication skills of nurses
Alternative treatment	•BCc1 nanomedicine •Cantharidin sodium •Jinlongshe Granule •Mistletoe treatment

## Discussion

The aim of this systematic review was to identify and summarize the influencing factors that may cause psycho-social burden and to highlight possible preventive interventions for patients with GC.

### Genetic Condition

The regulation of the hypothalamic-pituitary-adrenal (HPA) axis influences the biological aspect of stress response. Therefore, genetic factors affecting the HPA axis have an impact on the individual's HPA axis response and thus on the susceptibility to stress-related psychiatric conditions. Since FKBP5 is known as the major regulatory protein of the HPA axis it has received growing interest. Several studies reported FKBP5 as a promising gene target for anxiety and treatment response (71). Kang et al. described polymorphisms of FK506 binding protein (FKBP5) that are potent predictive factors for anxiety and depression after prolonged stress exposure due to treatment of GC. These findings led to the assumption that FKBP5 gene variants contribute to dysregulated HPA axis responsivity and lead to susceptible phenotypes such as depression and anxiety in light of long-lasting stressors (18). However, the study is limited by its small sample size. Moreover, Kang et al. investigated the impact of clinical phenotypes on stress but did not measure physiological stress responsivity, such as cortisol reactivity, and changes in the immune system. To confirm the genetic influence of FKBP5 gene polymorphisms on psychiatric morbidity following prolonged stress further research with a larger sample should be performed.

On the other hand, Koh et al. explored the association between the brain-derived neurotrophic factor (BDNF) Val66Met polymorphism and coping mechanisms on stress in patients with GC (17). Coping mechanisms are known to have an enormous effect on the development of psychological distress in response to external stressors (72). Thus, a better understanding of coping responses to cancer may improve medical care and QOL for patients with GC. BDNF influences neuronal proliferation and differentiation. Furthermore, BDNF is associated with neuronal plasticity in response to stressful events (73). The BDNF Val66Met polymorphism is a functional single nucleotide polymorphism of the BDNF gene. The Met allele of BDNF Val66Met may play a role in less adaptive genetic disposition toward adverse experience (74). Additionally, the Met allele is associated with decreased activity-dependent secretion of BDNF compared to the Val allele (75). Koh et al. reported that Met carriers of BDNF Val66Met polymorphism might be predisposed for an anxious coping response in under conditions of GC. Coping mechanisms are known as the interactions between individual vulnerability and external stress levels. Thus, it is of enormous importance to control external stress levels when assessing individual vulnerability of coping responses (17). A limitation of this study is the sample size with 91 participants, which was relatively small. Additionally, other individual factors, such as personality traits, that may have an impact on coping response, were not measured. However, a BDNF genetic variant may critically affect coping styles to acute stress in patients which should be further investigated.

### Treatment Method

There are several treatments to cure, to prolong life and to relieve symptoms in patients with GC, such as surgery, radiation, and chemotherapy. Regarding surgery treatment, gastrectomy is one of the most frequently used types of operation in patients with GC. Gockel et al. evaluated the difference of QOL of patients with subtotal gastrectomy compared to patients with total gastrectomy. Irrespective of the fact, that the study has shown a higher QOL of patients with subtotal gastrectomy, the nutritional status did not result in a significant difference in score. Furthermore, weight loss and gastrointestinal symptoms (i.e., diarrhea, nausea) were significantly higher in patients with total gastrectomy. This leads to the assumption that patients with subtotal gastrectomy show a higher QOL than patients with total gastrectomy. The study results are limited by the fact that they are based on a selection of patients who have undergone regular tumor follow-up and were free of recurrence. Thus, it is a selected random sample that limits the generalizability of the findings (34). Similar results were found in a study by Davies et al. They reported that patients who had undergone subtotal gastrectomy show a higher QOL after operation than before surgery and 1 year- postoperative compared to patients who had undergone total gastrectomy (33). Unfortunately, a comparison between the QOL in patients of this study with other patients' is limited due to the use of an old questionnaire.

These results differ from the outcome of the cohort study of Goh et al. which reported no differences in QOL between patients with subtotal gastrectomy and patients with total gastrectomy. Irrespective of these results, the measuring of the parameters “dysphagia” and “eating restrictions” has shown a significantly higher score in patients with TG (35). Several other studies have shown better QOL in patients after SG than TG due to reduced dumping and esophageal reflux, with better food intake and weight gain (33, 76). Understanding the possible differences in QOL after TG and SG will not influence clinical decision making, it is still highly relevant for physicians to be informed about the respective advantages and disadvantages.

On the other hand, a study of Park et al. indicates no differences between laparoscopic proximal gastrectomy (LPG) and laparoscopic total gastrectomy (LTG). Due to health- related QOL scores, physical function of the LPG group was significantly worse. Both groups showed a deterioration in gastrointestinal symptom scale (a.g. nausea, vomiting, diarrhea, reflux etc.). Postoperative symptoms such as fatigue, dysphagia, and eating restriction persisted for 2 years after surgery. Moreover, LPG appears superior in preventing vitamin B12 deficiency compared to LTG (28). Other studies have shown conflicting results, e.g., LPG offered little benefit regarding postoperative QOL (77, 78). To fully point out the advantages of LPG, long-term results with a large number of patients should be evaluated in the future. Surgery and more precisely post-surgical complications result in a significant worsening of patients' health- related QOL (26). This can be caused by the problem that patients are not adequately conversant about the impact of surgery and the long-term changes in QOL (36).

The gastrectomy is not the only factor influencing patients' QOL in terms of surgery methods. Ikeguchi et al. described different QOL outcomes due to various reconstruction methods. They have shown a QOL improvement of the patients due to pouch-double tract method (PDT) after total gastrectomy (TG) in terms of their nutritional conditions compared with a more traditional Roux-en-Y reconstruction (RY). Thus, PDT will improve QOL of patients who underwent TG. Patients who underwent a PDT showed lower symptoms in terms of dumping and better postoperative food intake along with better body weight recovery. PDT reconstruction after TG might have the benefit not only from an interposed pouch but also from a double tract. Even though a sample size of 29 patients is small, PDT is a safe reconstruction method and improves the post-operative QOL of patients suffering from GC in comparison with RY (31). Additionally, postoperative advantages—body weight and nutritional status—of double tract reconstruction after TG were reported (79).

Moreover, Kim et al. indicated that vagus nerve-preserving distal gastrectomy (VPG) leads to less diarrhea and appetite loss at 3 and 12 months after surgery along with a higher QOL score compared with a conventional distal gastrectomy (CG). Additionally, the CG group reported a decrease of physical functioning and role functioning in comparison with the VPG group (30). Several procedures have been designed to improve postoperative QOL in patients that underwent gastrectomy. The essential concept is the preservation of the pylorus (PPG) or the vagus nerve to maintain gastric function. In a retrospective nationwide assessment study in Japan, Fujita et al. described that patients after PPG show superior results compared to patients that underwent Billroth-I-distal gastrectomy (DGBI) in terms of suppressing postoperative dumping symptoms, diarrhea and the need for additional food. Preservation of the celiac branch of the vagus nerve is recommended either in DGBI or PPG for EGC (29). A limitation of both studies is that results may vary by the surgeon. However, additional clinical trials with larger sample sizes and more diverse populations are necessary. In conclusion, both trials give rise to VPG as a good alternative to CG in early GC patients in terms of postoperative QOL.

Regarding laparoscopic surgery treatments, several studies indicate that laparoscopy-assisted distal gastrectomy (LADG) does not only lead to less pain and better recovery but also improves patients' QOL in terms of global health and patient functioning compared to the QOL observed in patients after open operation (25–27). More precise, Lee et al. reported that there is no difference in QOL between Roux-en-Y, Billroth I, Billroth II, and Braun anastomosis reconstruction, but a higher QOL score is reported in patients after laparoscopic operation compared to patients underwent open operation (27). Misawa et al.'s studies have shown that role, emotional, cognitive, and social functioning scores are higher in patients that underwent laparoscopic gastrectomy at 6 and 12 months postoperatively, but not in physical functioning. Symptom scales are better in patients with laparoscopic surgery after 6 months but not at 12 months (25). A strength of this study is the comparison of QOL measured at 1-, 3-, 6-, and 12-months follow-up after surgery. In summary, comparison of LADG to open distal gastrectomy in patients suffering from GC shows an improved QOL outcome in patients that underwent LADG.

However, a study of Lee et al. pointed out the contrary: patients who underwent LADG experience lower QOL compared to patients who underwent open distal subtotal gastrectomy after the early postoperative period and before achieving long-term survival. In other words, the study compared the QOL of patients after LADG and patients that underwent open distal gastrectomy after the early postoperative period and before reaching 5 years postoperatively. These results may be associated with the patients' expectation (24). There are several definitions of QOL (80). One definition is the gap between the patient's expectations and the reality (81). According to this definition of QOL, a patient shows a high QOL if the gap between his or her expectations and reality is small. More precisely, the QOL of patients who underwent LADG is not high, when their expectations are high.

Furthermore, Kim et al. reported that fast-track surgery in LADG in patients with GC leads to an improvement in immediate postoperative QOL and postoperative recovery. They found that fast-track surgery was superior to the conventional system in terms of fatigue, appetite loss, financial problems, and anxiety. Thus, we can assume that the fast-track recovery system reduced anxiety, and earlier recovery at home might support appetite and increase physical activity. These results led them to assume that fast-track surgery improves immediate postoperative QOL in patients with GC. In summary, they showed that fast-track surgery has several positive impacts on QOL of patients with GC by shortening the duration of preoperative fasting, controlling pain sufficiently without opioids, providing early ambulation and quickly advancing diet (23).

Additionally, Avery et al. published a prospective study, where they found that gastrectomy for cancer has an enormous decreasing effect on QOL but mostly recovers after 2 years (37). Although the study was prospective and longitudinal using validated disease-specific questionnaires, the sample size was small. On the other hand, Yu et al. reported the contrary. Their study indicates that during the 5-year postoperative period, QOL decreases due to gastrointestinal symptoms such as dysphagia, reflux symptoms, eating restrictions and diarrhea. Even though, several symptoms slightly decreased during the first postoperative year, the deteriorated physical functioning, role functioning, cognitive functioning, poor body image and fatigue remain to negatively impact the patients' QOL even after the 5-year post-gastrectomy period (36).

Another study aimed to discover possible differences in determinants of QOL before and after surgery for GC. Before surgery the main predictors are physical and emotional function, while after surgery fatigue and dyspnea are the most pronounced predictors of global QOL. A limitation of this study was low qualitative research interviewing, which is needed to explore what the patients were thinking while filling in the questionnaires. Nevertheless, this study showed that global QOL scores may be influenced by different aspects of health before and after resection (32). Regarding these statements, it is highly recommended that patients receive accurate health- related QOL information about the possible postoperative symptoms of surgery for GC.

Moreover, Zieren et al. reported that regardless of the surgical treatment method, postoperative QOL is affected mainly by somatic complaints and physical limitations. Compared with the preoperative results, QOL decreased on discharge from hospital but was restored during the following 6 months in patients which remained disease-free (38). A limitation of this study is the sample size of 71 patients, which might be too small to make a reliable statement. Besides, a more extensive study with a total of 1,335 patients with GC also showed that cancer recurrence caused a significant deterioration in patients' QOL (82). Therefore, we can assume that cancer recurrence might be the decisive factor in impacting on patients' QOL.

Chemotherapy (CT) is another treatment method often used to cure patients with GC. Park et al. reported about patients treated with second-line CT who showed an improvement in terms of global QOL, emotional function, cognitive function and symptoms. Surprisingly, there was no difference in QOL between responders and non-responders. Metastatic or recurrent GC is an incurable condition where the aim of treatment is to improve survival and to lower symptoms, despite a low rate of actual tumor response. Unfortunately, this study contained no control group (20). The findings of this study are different from other endpoints, e.g., the effect of treatment on tumor volume, and therefore provide important insight into the role of CT in the normal daily life of patients with incurable disease.

Another study showed a relationship between the number of CT cycles and the level of QOL. Patients who have undergone 3–5 CT cycles showed a higher QOL than patients with <2 cycles. This result led to the assumption that QOL is directly linked to cancer treatment procedure, i.e., CT. Since CT is associated with side effects such as nausea, vomiting, or loss of hair, encouraging patients to consider CT might play an enormous role in the treatment outcome and QOL of patients with GC. Furthermore, the study has shown a significant difference between patients' QOL with pain compared to those with no pain. A similar correlation was found regarding fatigue and QOL. The evaluation of the QOL questionnaire led the authors to assume that the most impairing problems of patients with GC are fear about the future, thinking about the disease and its consequences, impatience, and depression (19).

Kassam et al. published a clinical trial on patients treated with CT combined with radiotherapy to cure GC. They reported that global QOL on the social, role, emotional, nausea and fatigue scales showed a worsening at completion of radiation and simultaneously was associated with the CT dose. More precise, a higher CT dose was associated with poorer scores for the nausea and vomiting scale and for global QOL (22). However, data are lacking on the relationship between long-term toxicity and QOL after radiotherapy for GC. Toxicity and other therapy factors affecting patients' outcomes in the short and long term are important areas for future studies.

Furthermore, Cui et al. explored the combination of CT and immunotherapy on patients' overall QOL. Their cohort study indicates that CT combined with cellular immunotherapy improves patients' QOL and leads to a longer progression-free survival period compared with patients only treated with CT (21). A weakness of this study was the relatively short follow-up period and the small sample size. Although immunotherapy has gained attention in the treatment of cancer, it is not a routine treatment for patients with GC compared with surgery and CT due to the limited efficacy and only few clinical trials. However, other studies proposed that immune imbalance was not altered in patients with early cancer but was affected in patients with advanced cancer (83). This finding suggests that immunotherapy offers a greater survival benefit for patients with advanced cancer.

On the other hand, Kim et al. reported an only slight improvement in a few QOL domains such as functioning and global health when comparing changes in QOL according to tumors' response to first-line palliative chemotherapy. These results indicate that overall QOL was not affected in patients whose tumors were not shrunk compared to those who were. This observation led them to assume that patients' QOL might be constant, regardless of the tumor's response, if the tumor is effectively controlled by first-line palliative CT. A limitation of this study is a deterioration of participants' compliance with completing the QOL questionnaires from 95.1 to 75.8% (4). In future studies there should be a careful monitoring of patients' compliance with QOL questionnaires until the end of the study to assess comprehensive QOL outcomes.

### Blood Markers

The study of Xu et al. has shown that patients with anxiety or depression were found to have a significantly higher preoperative neutrophil-lymphocyte ratio (NLR), assuming that NLR was not only related to the prognosis of gastric cancer as previously shown (84), but was also linked with preoperative anxiety and depression in patients with GC (10). Additionally, it was reported that the increase of proinflammatory cytokines in the peripheral blood of patients with cancer is linked with the development of depression (85). Therefore, NLR should be actively monitored also in order to early on consider anxiety and depression in patients with cancer. The present results of this study should be confirmed by a multicenter trial containing a large sample size, since this study was limited by a small sample size and a simple methodology (single-center study) (10).

Fujita et al. explored the impact of circulating-alpha-2-macroglobuline (A2M) levels on mental status of patients with GC. Their study compared A2M serum levels of depressed patients with those of non-depressed patients. The main function of A2M is the irreversible covalent capture of proteinases and the endocytosis of these complexes. Besides, A2M regulates the biological activity of a variety of cytokines (86). In this study serum levels of A2M were significantly elevated in patients who had undergone TG or SG. The reason for the elevation of A2M in patients with gastrectomy is not clear yet. Moreover, their study found that circulating A2M elevation plays a role in the development of postoperative depression in patients with GC. In other words, depressed patients showed significant higher A2M serum levels than non-depressed patients (40). Higher levels of circulating A2M may be used as an additional marker of clinical depression in postoperative patients, but further studies are needed with a large sample to clarify the possible link of A2M elevation with the development of depression.

Moreover, Arner et al. reported about cancer patients with a lower serum level of circulating Carnosine Dipeptidase 1 (CNDP1), which tend to have a higher weight loss, malnutrition and poor QOL (39). They explored specific alterations in protein levels associated with body weight/fat mass loss in patients with GC with cachexia compared to weight-stable patients. This procedure identified a lower CNDP1 concentration in patients suffering from greater weight loss. CNDP1 is a protein, which is known for being reduced in metastatic prostate cancer and glioblastoma (87). Arner et al. pointed out that CNDP1 levels were negatively correlated with weight loss but positively associated with plasma albumin, IGF1, and QOL. Additionally, there was a significant positive correlation between CNDP1 and survival in patients with cancer (39). Since the functional role of CNDP1 remains unclear, the actual prognostic value of CNDP1 needs to be validated in larger future studies.

In addition, Correia et al. performed a study with the main finding that cytokines, more precisely TNF-alpha, might be specifically associated to malnutrition along with worse QOL in patients with GC. QOL assessment identified anorexia and pain as the main symptoms associated with worsening of QOL. However, the small number of patients included might considerably reduce the statistical power of this study (42). Nonetheless, multifactorial in its etiology, malnutrition may result from a complex interaction of major central nervous system and metabolic abnormalities attributable to a combination of tumor side product interactions and cytokine release (88). Thus, measurement of TNF-alpha serum levels could help physicians not only to identify malnourished patients in a more efficient way but would also help to select patients which might benefit from a nutritional intervention.

Pan et al. published a clinical trial reporting that both serum and tissue leptin-LepRb are higher in depressive than in non-depressed patients with GC (41). Leptin is a hormone primarily produced in white adipose tissue and secreted into the plasma. It is known for controlling food intake and energy balance *via* its receptor (LepRb). Several clinical studies reported that elevated serum leptin levels might serve as a biological marker and predictor for depression (89). Due to these findings Leptin-LepRb might be a potential diagnostic marker and therapeutic target in patients with GC suffering from depressive disorders.

Furthermore, Huang et al. assumed that reactive oxygen species (ROS)-activated ABL1 plays a role in the development of depression in patients with GC. Specifically, patients with GC and depression showed a high level of oxidative stress and dysregulated inflammation compared to non-depressed patients with GC. They assumed that ROS may regulate the expression of ABL1 by influencing receptor tyrosine kinases, which lead to the phosphorylation of ABL1. Additionally, ABL1 was found to regulate the inflammatory signaling pathway. The results of this study confirmed that ABL1 was expressed significantly higher in patients with GC and depression which leads to a higher activity of the inflammatory pathway along with an increased risk of depressive disorders (43). These findings are corresponding with a study of Wei et al. which also reported that oxidative imbalance and oxidative stress in depressed patients with GC may influence the onset and exacerbation of depression. More specific, depressed patients showed significantly lower serum levels of catalase and superoxide dismutase concentrations along with a decreased antioxidant and antisuperoxide anion capacity, while serum malondialdehyd levels were higher compared to non-depressed patients with GC. Thus, Wei et al. assumed that the etiology of depression involves free oxygen radicals and their derivatives. In other words, oxidative stress might play a role in the pathophysiology of depressive disorders (44). Therefore, the results of this study may have an enormous impact on pharmacological interventions targeting cellular antioxidants since this may be a promising strategy against oxidative imbalances in depressed patients with GC. More detailed research is needed to clarify the exact role of oxidative stress in depressive disorders.

### Nutritional Status

Unintended weight loss is often seen in patients with gastrointestinal cancer at diagnosis (90). Malnutrition and weight loss may influence QOL and survival. Therefore, QOL may be improved by changing the nutritional status (91). Tian et al. confirmed that a higher daily nutrition intake in patients with GC is positively correlated with QOL. Most of the patients with GC showed lower daily nutrition intake than the reference groups. The factors influencing daily nutrition intake of the participants were number of meals a day, family income, way of operation, physical exercise, and age (45). Park et al. came to a similar conclusion. They indicate that there might be a significantly difference in patients' QOL depending on the BMI shift after TG. In other words, patients with a significant lower BMI after surgery show a worse score in terms of gastrointestinal symptoms, pain, reflux symptoms, and body image (46). Again, this was confirmed by Climent et al. (47). Their results emphasize that patients with >10% body weight loss (BWL) after 2 years are suffering from more gastrointestinal symptoms compared to patients with less BWL. Furthermore, patients losing at least 10% of weight at 2 years experienced preoperatively more fatigue, pain, and constipation along with a significant reduction in role and physical function as compared to patients with a lower BWL (47). A weakness of the study is the lack of a multivariate analysis taking confounders into account to identify predictive factors for long-term BWL after surgery. A multi-center study in the future would provide more generalizable data.

Regarding post-surgery nutrition, Hur et al. performed a randomized clinical trial, which states that early oral feeding after GC surgery may result in shorter hospitalization and may improve the QOL of patients in terms of less fatigue and less nausea in the early postoperative period. The patients in the early feeding group were started on sips of water on the first postoperative day and began on a soft diet on the third postoperative day, while patients in the control group began with sips of water on the third postoperative day and a soft diet on the sixth postoperative day. In conclusion, they showed that patients who got early enteral feeding had a better metabolic response and a shorter hospital stay compared with patients in the control group (48). Since this trial was conducted at a single center and was limited by the sample size, a multi-center trial may provide more clinical evidence supporting early oral feeding after GC surgery. On the other hand, Jin et al. reported in their study that post-surgical parenteral nutrition may improve the nutritional and psychological status, QOL and immune function of patients with GC after surgery. Even though there was no difference in QOL assessment comparing the study and the control group, there was a significant difference in the HADS assessment. These results indicate that parenteral nutrition not only improves the nutritional status of patients with GC that underwent gastrectomy, but also provides benefits in terms of increased psychological status along with QOL. Regarding the impact on immune functions, the study group showed an increase of CD^3+^ and CD^4+^ after parenteral nutrition compared to those in the control group. Additionally, there was a significant higher ratio of CD^4+^/CD^8+^ in the study group in comparison to that in the control group (49). Although the effects of parenteral nutrition on markers of nutritional status were measured, there was no evaluation of changes in physiological factors such as body weight and BMI, that might affect the patients' health state. Since it is well-known that malnutrition results in immunosuppression and deterioration of patients' QOL, appropriate clinical and institutional approaches and active medical interventions are required for improving patients' nutritional status after surgery.

### Daily Living

In the last decade, several studies were conducted regarding patients' daily living in terms of influencing factors on overall well-being and global QOL in patients suffering from GC. Tian et al. showed that the patients' residence area plays a role in their QOL. They compared the QOL of 162 patients living in an urban county with 200 patients living in a rural city. Apparently, the QOL of rural living patients is worse compared to the QOL of urban residing patients. They assumed that rural living patients reported more physical and psychological pain due to their lower levels of education and family income compared with the urban residing patients. Additionally, the authors pointed out that enhanced nutrition and rehabilitating exercise positively influence the QOL of patients with GC (50). Similar results referring to the patients' income were reported by Jeong et al. The results of their study led them to assume that older patients and patients with less income felt more depressed, while patients with less income and patients with less social support felt more anxious. More specifically, they showed that social support may lower the psychosocial burden of the lack of monetary resources in the treatment process (56). In conclusion, social support, and patients' income may have an influence on perceived anxiety of patients with GC. With a set of 52 patients, it is difficult to generalize the findings of this study.

Two reported a link between QOL of patients with GC and their educational background (51, 54), while another study pointed to the opposite (19). Higher educated patients tend to do their own research regarding their health status which may have a negative effect on their QOL due to psychological strain caused by the severity and seriousness of the disease. There are only few studies on QOL in patients with GC using the 15D instrument, which was used in this study. Compared to the frequently used questionnaires, e.g., the EORTC QOL-C30, the 15D instrument can be answered by more patients with different educational status in a shorter period. Moreover, there are factors such as “sexual activity” which are not covered by other questionnaires. Irrespective of this fact, further studies with a higher level of participants utilizing a combination of generic and specific tools are needed to provide a more detailed and more precise assessment of patients' QOL.

A similar result was published by Kim et al. (52). They indicate that a lower education level and a higher disease stage are associated with psychological distress in patients with GC, while psychological distress might be associated with worse outcomes. More specifically, patients with psychological distress tend to have worse disease-free survival and poorer overall survival than those without psychological stress (52). However, there are several limitations in this study: First, some patients filled in the questionnaires after receiving the prognosis of their disease, while others did when not knowing their prognosis or treatment plan. Second, it is possible that patients may have under- or overestimated their status since the questionnaires were filled in by self-report. Third, the CT of the patients was not standardized.

Not only the general education influences QOL in patients suffering from GC, but also the specific education in terms of the disease. This assumption was published by Faller et al. They compared the impact of an interactive, patient-oriented group program with a lecture-based information program in patients with GC. This prospective, controlled study pointed out that patients attending an interactive education program regarding information about their disease had more knowledge, showed more active coping, and reported a better QOL (53). Limitations of this study are the relatively small sample size of 121 participants and differential durations of the interventions.

The outcomes of the study of Dang et al. emphasize the importance of certain therapeutic interventions to improve patients' QOL. Since “sexual activity” was the dimension with the lowest score along with “discomfort and symptoms,” certain therapeutic interventions may induce an improvement of the patients' QOL in both clinical practice and healthcare management (54). Furthermore, the discovered health- related QOL score of patients with GC was higher compared to patients suffering from other cancer types for instance lung cancer, colorectal cancer, or breast cancer (92). It can be assumed that this outcome was due to the fact that the major number of participants showed stable stages of GC. The fact that the study has shown sexual impairment as the dimension measured with the lowest score is in line with other studies showing similar results (93). Therefore, improving “sexual activity” might be highly important for patients with GC. Additional lower-scored factors were “usual activity” and “discomfort and symptoms” (54), which suggests an affectation of daily living in patients with GC. Furthermore, Hofheinz et al. reported that patients with GC treated with CT consider not only a survival benefit *per se*, but as more important a survival benefit accompanied by high QOL in terms of being able to self-care and receiving a treatment with good tolerability. In the qualitative interviews the “ability to self-care,” “treatment tolerability,” and “survival benefit” seem to be the key factors in influencing patients' overall well-being. In the quantitative interviews, when directly asking the patients about additional treatment goals, 27.3% of them mentioned “to experience no limitations in daily routine” (55). The findings led them to assume that patients with previous CT experience consider a survival benefit accompanied by high QOL, i.e., being able to self-care and receiving a treatment with good tolerability, as more important than only an additional survival benefit. Tumor response, performance status, and toxicity data were not captured, which might be a limitation of this study. Another study investigated the effect of nursing care given at home on the QOL of patients with GC. The pre-test QOL of patients in the experimental and control group were reported to be below average. Nursing care provided during the home visits to the patients in the experimental group was found to improve patients' overall well-being and QOL. Symptoms such as fatigue, vomiting, sleep difficulty, and loss of appetite improved due to nursing care given at home (2). Other studies also reported that an accurate and effective symptom management might positively impact the patients' outcome (94). A limitation of the study was the longer stay at the hospital of the control group. Nevertheless, the statistical evaluation of the EORTC-QLQ-C30 fulfilled by the patients pointed out that longer stays at the hospital positively impact patients' QOL by making it more uncomplicated for these patients to attend their treatments (e.g., CT cycles) (2). In light of these results, it can be suggested that the arrangement needed for homecare of patients with GC should be planned and implemented early after the treatment at the hospital.

### State of Health

In 2018 Hu et al. performed a clinical trial including 57,506 participants in Taiwan, which reported female sex and hypertension as predictive variables for the development of a depression among patients suffering from GC. Another main finding of this study was that the risk of depressive disorders was higher among patients with GC compared to patients without GC (57). Since GC is often diagnosed at a late stage and its survival prognosis is often poor (95), patients with GC may have a higher risk of experiencing psychological distress. Moreover, different types of surgery treatment may affect nutritional status and physical and emotional functioning. From a psychosomatic point of view, hypertension could be concomitant with depressive disorders (96). Depression might influence the occurrence and development of hypertension (97). Furthermore, females were at greater risk for developing depressive disorders. This result is consistent with previous studies which also reported female sex as a risk factor for depression in patients suffering from cancer (98).

On the other hand, acute muscle wasting seems to negatively affect the QOL of patients with GC. More precisely, patients with muscle wasting >10% tend to show a poorer QOL in terms of fatigue and physical functioning postoperatively, along with a higher risk of postoperative complications and longer hospital stays (58). A main limitation of this study is that the measurement of muscle mass was not performed beyond 1 week post- surgery. Furthermore, QOL assessment no longer showed significant differences between both groups 6 months after surgery, while it is unknown whether muscle mass recovered simultaneously. To clarify these questions, future multi-center studies with muscle mass assessments beyond 1 week after surgery are needed. However, the results emphasize that prevention of postoperative muscle wasting can be a promising strategy to improve QOL after surgical treatment of GC.

### Mental State

Little is known about the possible predictors of postoperative QOL and anxiety symptoms of patients suffering from GC. One of these potential predictors could be emotional competence (EC), which has shown a positive impact on adjustment to cancer (97). According to the study of Baudry et al., EC appears to be a decent predictor of emotional distress of patients with GC. When it comes to anxiety and depression symptoms, patients with GC report higher levels than patients with other types of cancer. Furthermore, patients who tend to oftentimes use their EC in daily life experienced fewer anxiety and depression symptoms and showed higher health- related QOL shortly after diagnosis. Due to their EC, those patients might be more efficacious in controlling the emotional impact of the diagnosis and surgery, such as in restricting negative emotions (12). To evaluate more comprehensive results, future studies should take into account a better homogeneity in the delay between surgery and questionnaire completion. How people cope with cancer diagnosis and how they deal with the situation can have an enormous impact on their health status, regardless of the type of cancer. The development of the patients' overall QOL not only depends on the treatment level, but also is contingent on patients' attitudes toward their disease (99). Since the measurement of QOL is influenced by subjective judgements of perceived qualities, it is very likely that personality has an impact on QOL. Therefore, another possible predictor for anxiety or depression in patients with GC might be one's type of personality. A study confirms the assumption that the Type D personality might be associated with a deteriorated QOL. Furthermore, the authors assumed that Type D personality is a general vulnerability factor in terms of lower QOL across populations (100). Depressed patients with cancer may experience more severe symptoms. The results of the study of Zhang et al. highlight the fact that patient personalities are linked to overall survival QOL in Chinese patients with GC (60). However, this study contains weaknesses, e.g., the non-randomized design and the lack of access to patient files of the non-responders.

Regarding patients' personality type associated with QOL, Yamaoka et al. reported about the tolerant type, which tends to show a greater health- related QOL score compared to the intolerant type. Patients' personality type was evaluated using the Eysenck Personality Questionnaire. Their results led them to the assumption that the personality type is a possible mediating factor modulating the health- related QOL-20 questionnaire responses followed by the outcome measure, health-related QOL (59). On the other hand, a study confirmed that in patients with GC, adaptation after gastrectomy shows the strongest impact on patients' QOL. Simultaneously, social support showed to have the greatest influence on the adaptation of patients with GC, followed by anxiety, spiritual well-being and gastrointestinal symptoms. The study verified that adaptation to the diagnosis of GC has an enormous impact on patients' QOL (15). Through successful adaptation, patients can manage not only the associated physical symptoms but also their social changes to nearly keep their regular roles, e.g., as parents. This allows them to actively continue their daily lives and manage their emotions (101). These changes are ultimately affecting QOL in a positive manner.

Moreover, several studies reported an increased risk for psychological distress in patients with cancer who had experienced psychological disorders before their cancer diagnosis (102). Nordin et al. emphasized patients' level of depression and anxiety at diagnosis as a predictor of depression and anxiety 6 months later. A multiple regression analysis showed that the only additional risk factor at diagnosis besides anxiety and depression was advanced disease and a lack of somebody besides the family to rely on (61). These assumptions are consistent with a study of Ben-Ezra et al. which concluded that patients with GC who experienced previous trauma showed higher depressive symptoms, lower perceived social support, and lower future life satisfaction relative to patients without previous trauma. Additionally, gender and marital status were predictors of psychiatric symptoms, while age, marital status, and past life satisfaction were predictors of well-being (62). The study used a cross-sectional design while a longitudinal one might be desirable for the future. Moreover, they used self-report indices while a clinical interview could have helped in diagnosing psychiatric disorders or previous trauma. Additionally, since the sample size is relatively small, it is difficult to generalize the findings of this study.

However, also the mental health status of patients with GC should be considered in terms of the length of the waiting period for cancer treatment. Even though psychological consequences regarding the length of waiting time for treatment among cancer patients are still largely unknown, the findings of Song et al. considering the relation between waiting time and new onset of mental disorders in patients with GC affirm the results of other studies including breast cancer patients (103). This study highlights the assumption that patients without any pre-existing mental disorder benefit from longer waiting times for cancer treatment in terms of new onset of a mental disorder whereas patients with a previous history of mental disorders showed an increased risk of aggravation regarding their mental disorder. A deterioration can be prevented by quick treatment decisions (63). A major strength of this cohort study is the huge sample-size. However, the study is limited by the lack of data on the exact starting date of cancer treatment.

### Supportive Care

Communicating efficacious in delivering bad news is an important skill for nurses, especially in oncology. Nevertheless, numerous oncology nurses experience difficulties in communicating, for instance in supporting patients lately given bad news such as a cancer diagnosis (104). A study of Fukui et al. showed that improved communication skills by nurses were found to lower psychologic stress among patients after being informed of a cancer diagnosis. In other words, care by nurses who completed the communication skill training was reported to decrease psychological distress and improve coping style in patients informed of their cancer diagnosis (66). A similar statement was published by Fukui et al. The main finding of this randomized clinical trial was that communication skills training of nurses seems to improve patients' satisfaction with healthcare professionals and QOL just after being diagnosed with GC (64). The generalizability of both findings remains uncertain. Only one institution was recruited, and the sample was small. Moreover, the way each physician and nurse performed the interview may have affected the patients' outcome. Further studies with larger sample sizes in a multi-center design are needed.

Furthermore, in 2020 Rha et al. reported that one of the main unmet needs of patients with GC seems to be insufficient health system and information domain. More precisely, the most unmet needs items with “Having one member of hospital staff with whom you can talk about all aspects of your condition, treatment, and follow-up,” “Having access to professional counseling if you, family, or friends need it” and “Being informed about things you can do to help yourself to get well” belonged to the health system and information domain. Besides, physical, and daily living unmet needs, depression, and symptom severity directly affect patients' QOL as well. In other words, physical and daily living unmet needs as mediators have an impact on the influence of symptom experience on patients' QOL (65). These data clearly point toward the need for professional psychooncological support for hospitalized patients but also those in an ambulatory setting. The benefit of this support should be investigated in a larger future randomized clinical study in patients with GC.

### Alternative Treatment

For some patients with stage IV GC, surgery is not an option anymore due to distant metastases. Here, CT is the leading palliative treatment. However, with the increasing problem of tumor drug resistance and due to immune suppression-related problems, many patients are unable to withstand or to complete CT as scheduled, which directly affects survival and QOL (105). In such cases, the possibility to use alternative treatment methods may be of great advantage for these patients.

One of these alternatives was reported by Kim et al. in their randomized clinical study about the possible effects of mistletoe extracts, more precisely Viscum album, in the treatment of patients with GC (70). It is known that surgical stress shows a suppressing effect on the immune system, e.g., a decrease of granulocyte function, numbers of natural killer cells and T-helper lymphocytes after major surgery (106). Mistletoe extracts were reported to have immunomodulatory effects by enhancing the secretion of cytokines and the activity of immunological effector cells (107). Kim et al.'s study pointed out that adjuvant mistletoe treatment in patients with GC may lead to less frequent diarrhea and might be associated with improved QOL. They used an injectable, endotoxin-free plant extract from the European mistletoe species Viscum album L. White blood cell count and eosinophils occurred to be elevated in the patients treated with oral CT combined with mistletoe extract compared to the control group. Moreover, these patients showed a improved global health status (70). Due to the relatively small sample size of this trial, further research with greater sample sizes is needed.

On the other hand, Sun et al. published a double-blind placebo- controlled clinical trial in 2015 about the additional use of Chinese medicine, more precisely Jinlongshe Granule, combined with other Chinese medicine and its positive impact on the somatic function, role function, emotional function, social function, cognitive function, and general QOL of patients with GC stage IV (69). Jinlongshe Granules mainly consist of Arisaematis Rhizoma Preparatum, Pinelliae Rhizoma Preparatum, Cremastrae Pseudobulbus, and Paridis Rhizoma. It is widely used in military hospitals in China for over more than 10 years. Sun et al. showed that Jinlongshe Granule might lower the symptoms of fatigue, nausea and vomiting, pain, loss of appetite, and constipation in patients suffering from advanced GC (69). Several studies showed that Jinlongshe Granule might inhibit gastric cancer progression from multiple perspectives and aspects, e.g., by disturbing the DNA synthesis and decrease of proliferous activity of cancer cells by inhibiting the expression of proliferating cell nuclear antigen (PCNA) and epidermal growth factor receptor (108). However, studies outside of China are missing so far.

In 2012 Zhan et al. reported in a clinical study about clinical benefit, QOL and side effects of chemotherapy in patients with GC by using a Qinin injection combined with chemotherapy (68). Qinin (Cantharidin sodium) is a semi-synthetic derivative of cantharidin and is a Chinese herbal preparation with proposed anti-cancer activity. It is used as an alternative additional treatment of solid tumors including GC. The main active ingredient cantharidin shows anti-cancer characteristics without causing myelosuppression. Moreover, it might stimulate hematopoietic stem cells to differentiate into myelomonocytic cells along with an increase of leukocytes (109). Even though, Zhan et al.'s study did not show a statistically significant effect regarding short-term efficacy, a clinical benefit in patients with GC was shown. Thus, Cantharidin sodium injection in combination with CT might lower side effects caused by CT along with an improvement of QOL (68). To clarify the possible effects of this additional therapy further clinical trials with randomized groups are needed.

Regarding the effects of nanomedicine, Hafizi et al. published a randomized, double-blind, placebo-controlled investigation where they indicate that BCc1 nanomedicine might improve patients' QOL and leads to higher overall survival by reducing CD44 expression in tumor cells (67). BCc1 contains chelating iron which can stop the G1/S phase along with causing death of cancer cells by using apoptosis mechanisms. Surprisingly, there was no apoptosis seen in normal cells with an identical BCc1 dose, while apoptosis was induced in cancer cells (110). Additionally, they showed that BCc1 might have protecting effects against oxidative stress in patients with. Nanomedicine may provide new approaches regarding advanced cancer treatment. Further studies in the future hopefully confirm the effectiveness of nanomedicine in cancer treatment.

### Strength and Limitations

There are several limitations to this systematic review as a systematic review is evidently dependent on the quality of the studies under review. The number of suitable studies was moderately small, as was the number of patients in most cohorts. Furthermore, the instruments applied were different in most of the studies and therefore not comparable or transferable with each other. Nevertheless, a strength of this systematic review was the wide database screening accompanied by the identification of several various influencing factors. Additionally, there has been no exclusion of studies regarding their year of publication.

## Conclusion

GC is a complex disease and there are several factors which affect patients' well-being and may lead to psychosocial burden. Furthermore, psychosocial burden may cause a versatile impairment that may not be comprehensively measured with a single questionnaire. It has been indicated that QOL is affected by the emotional, social, and physical aspects of a disease and its treatment, in other words how it is experienced and the extent to which it affects the life of a patient. QOL is acknowledged to play an enormous role in patients' oncologic outcomes and overall well-being. As an early predictor of disease progression, assessing QOL could help physicians to monitor the patients more closely. Clinical decisions should be patient-centered and made with more consideration of the potential advantages and disadvantages for patients' overall well-being as discussed above. In summary, preventing psychosocial burden by improving patients' QOL e.g., by—besides choosing the right somatic treatment—offering professional psychooncological support should be as important as the survival benefit that any treatment may provide.

## Data Availability Statement

The original contributions presented in the study are included in the article/supplementary material, further inquiries can be directed to the corresponding author/s.

## Author Contributions

SR performed the systematic search and wrote the first draft of the manuscript. AS planned the study and gave critical input throughout the study. All authors finalized the manuscript.

## Conflict of Interest

The authors declare that the research was conducted in the absence of any commercial or financial relationships that could be construed as a potential conflict of interest.
